# Bacterial *versus* fungal laccase: potential for micropollutant degradation

**DOI:** 10.1186/2191-0855-3-63

**Published:** 2013-10-24

**Authors:** Jonas Margot, Chloé Bennati-Granier, Julien Maillard, Paqui Blánquez, David A Barry, Christof Holliger

**Affiliations:** 1 Laboratory for Environmental Biotechnology, School of Architecture, Civil and Environmental Engineering (ENAC), Station 6, Ecole Polytechnique Fédérale de Lausanne (EPFL), 1015 Lausanne, Switzerland; 2 Ecological Engineering Laboratory, School of Architecture, Civil and Environmental Engineering (ENAC), Station 2, Ecole Polytechnique Fédérale de Lausanne (EPFL), 1015 Lausanne, Switzerland; 3 Departament d’Enginyeria Química, Escola d’Enginyeria, Universitat Autònoma de Barcelona (UAB), 08193 Bellaterra, Spain; 4 Present address: Unité de Biotechnologie des Champignons Filamenteux, Institut National de le Recherche Agronomique (INRA)/Universités de Provence et de la Méditerranée, Ecole Supérieure des Ingénieurs de Luminy, 163 avenue de Luminy-CP 925, 13288 Cedex 09, Marseille, France

**Keywords:** Laccase, *Streptomyces* spp, *Trametes versicolor*, Micropollutant, Wastewater, Oxidation

## Abstract

Relatively high concentrations of micropollutants in municipal wastewater treatment plant (WWTP) effluents underscore the necessity to develop additional treatment steps prior to discharge of treated wastewater. Microorganisms that produce unspecific oxidative enzymes such as laccases are a potential means to improve biodegradation of these compounds. Four strains of the bacterial genus *Streptomyces* (*S. cyaneus*, *S. ipomoea*, *S. griseus* and *S. psammoticus*) and the white-rot fungus *Trametes versicolor* were studied for their ability to produce active extracellular laccase in biologically treated wastewater with different carbon sources. Among the *Streptomyces* strains evaluated, only *S. cyaneus* produced extracellular laccase with sufficient activity to envisage its potential use in WWTPs. Laccase activity produced by *T. versicolor* was more than 20 times greater, the highest activity being observed with ash branches as the sole carbon source. The laccase preparation of *S. cyaneus* (abbreviated L_*Sc*_) and commercial laccase from *T. versicolor* (L_*Tv*_) were further compared in terms of their activity at different pH and temperatures, their stability, their substrate range, and their micropollutant oxidation efficiency. L_*Sc*_ and L_*Tv*_ showed highest activities under acidic conditions (around pH 3 to 5), but L_*Tv*_ was active over wider pH and temperature ranges than L_*Sc*_, especially at near-neutral pH and between 10 and 25°C (typical conditions found in WWTPs). L_*Tv*_ was also less affected by pH inactivation. Both laccase preparations oxidized the three micropollutants tested, bisphenol A, diclofenac and mefenamic acid, with faster degradation kinetics observed for L_*Tv*_. Overall, *T. versicolor* appeared to be the better candidate to remove micropollutants from wastewater in a dedicated post-treatment step.

## Introduction

Many micropollutants present in municipal wastewater, such as pharmaceuticals or biocides, are not easily removed in conventional biological treatments, resulting in a constant input into the aquatic environment (Deblonde et al. 2011; Margot et al. 2013a). As these compounds are designed to be biologically active, they can affect sensitive aquatic organisms even at low concentrations (Alan et al. 2008; Bundschuh et al. 2011; Gagné et al. 2011). One potential means to reduce the amounts released to the environment is to improve their biodegradation in a post-treatment step using microorganisms that produce oxidative enzymes such as laccases (Blánquez et al. 2008; Zhang & Geißen 2012).

Laccases (EC 1.10.3.2) are polyphenol oxidases that catalyse the oxidation of various aromatic compounds, particularly those with electron-donating groups such as phenols (−OH) and anilines (−NH_2_), by using molecular oxygen as an electron acceptor (Gianfreda et al. 1999). Laccase enzymes are widespread among plants, fungi and bacteria, and have various biological functions, such as degradation of complex polymers (lignin, humic acid), lignification, detoxification, pathogenicity, morphogenesis, sporulation, polymerization of melanin and spore coat resistance (Strong & Claus 2011). The ability of fungal laccases to catalyze (alone or with the help of mediators) the oxidation of pharmaceuticals and biocides was shown for several substances, such as endocrine compounds (Auriol et al. 2008; Cabana et al. 2007), analgesic and anti-inflammatory drugs (Lu et al. 2009; Marco-Urrea et al. 2010; Margot et al. 2013b), antibiotics (Schwarz et al. 2010; Suda et al. 2012), UV filter (Garcia et al. 2011), biocides (Margot et al. 2013b) and various halogenated pesticides (Torres-Duarte et al. 2009). Due to their wide range of substrates and the sole requirement of oxygen as the co-substrate, laccases appear to be a promising biocatalyst to enhance the biodegradation of micropollutants in wastewater in a complementary treatment step.

In order to overcome the cost associated with the large amount of free laccase required in real applications (due to losses during the treatment), two strategies have been envisaged, i.e., (i) immobilization of the enzymes on solid supports in order to reuse them several times (Fernández-Fernández et al. 2012) or (ii) production of the enzyme during wastewater treatment using laccase-producing microorganisms and cheap substrates (e.g., agriculture or forestry waste) (Libra et al. 2003). The latter option avoids expensive immobilization processes while it could further improve the degradation of micropollutants along with other oxidative enzymes produced by these organisms, such as peroxidases or oxygenases. It would, however, require growing and maintaining the laccase-producing organisms in the wastewater treatment plants (WWTPs), a process that is still little studied (Zhang & Geißen 2012; Libra et al. 2003; Blánquez et al. 2006). While the extensively studied white-rot wood-degrading fungi such as *Trametes versicolor* are attractive candidates with their high production rates of extracellular lignolytic enzymes (Nyanhongo et al. 2007), very little is known about the potential of bacterial laccases for bioremediation applications. Wastewater treatment involving bacteria is, however, considered to be more stable, as bacteria generally tolerate a broader range of habitats and grow faster than fungi (Harms et al. 2011). Moreover, in contrast to fungal laccases, some bacterial laccases can be highly active and much more stable at high temperatures, at high pH as well as at high chloride concentrations (Bugg et al. 2011; Dwivedi et al. 2011; Reiss et al. 2011; Sharma et al. 2007).

Most bacterial laccases studied so far are located intracellularly, which is a disadvantage for micropollutant degradation (Sharma et al. 2007). However, some strains of *Streptomyces* spp. produce extracellular laccases, such as *S. psammoticus* MTCC 7334 (Niladevi et al. 2008a), *S. cyaneus* CECT 3335 (Arias et al. 2003), *S. ipomoea* CECT 3341 (Molina-Guijarro et al. 2009) or *S. griseus* NBRC 13350 (Endo et al. 2002). Moreover, laccases from *S. psammoticus* and *S. ipomoea* showed unusually high activity at the slightly alkaline pH values (7–8) found in wastewater, as well as tolerance to high NaCl (> 1 M) concentrations (Molina-Guijarro et al. 2009; Niladevi et al. 2008a). High laccase activity was also observed in the culture supernatant of *S. psammoticus* and *S. cyaneus* (Arias et al. 2003; Niladevi et al. 2009), suggesting suitability of these strains for bioremediation applications.

The goal of this study was thus to assess the potential of four laccase-producing strains of *Streptomyces* bacteria, namely *S. cyaneus* CECT 3335, *S. psammoticus* MTCC 7334, *S. ipomoea* CECT 3341, and *S. griseus* NBRC 13350, together with the white-rot fungus *T. versicolor*, to select the best candidate for future use in municipal wastewater post-treatment, e.g., in a biotrickling or sand filter. More specifically, the goals were to study: (i) their ability to produce laccase in biologically treated wastewater on cheap substrates, such as agricultural, forestry or food industry wastes, in a sufficient quantity to oxidize the pollutants in a reasonable time (< 1 d), (ii) their laccase activity at different pH and temperature in order to determine optimal conditions for wastewater treatment, (iii) the inhibition of laccase activity by compounds present in wastewater such as salts, (iv) laccase stability in the pH range potentially found in the treatment, and finally (v) the laccase substrate range and their ability to oxidize different phenolic and aniline micropollutants in the pH range found in wastewater.

## Materials and methods

### Chemicals, choice of micropollutants, and commercial laccase enzyme

Three micropollutants were selected as model compounds for this study because of their regular presence in municipal WWTP effluent at relatively high concentrations (average between 300–1000 ng l^-1^) (Kase et al. 2011), their potential toxicity (Crain et al. 2007; Triebskorn et al. 2004) and because they are prone to oxidation by the laccase of *T. versicolor* (Margot et al. 2013b): the anti-inflammatory drugs mefenamic acid (MFA) and diclofenac (DCF), both aniline compounds, and the plastic additive bisphenol A (BPA), a phenolic substance.

BPA, DFC sodium salt, and MFA (purity > 97%), laccase preparation from *T. versicolor* (ref. 38429, Sigma), 2,2'-azino-bis(3-ethylbenzthiazoline-6-sulphonic acid) (ABTS), 2,6-dimethoxyphenol (DMP), syringaldazine and guaiacol were purchased from Sigma-Aldrich Chemie GmbH (Buchs, Switzerland). All other chemicals used were purchased from either Sigma-Aldrich or Fisher Scientific AG (Wohlen, Switzerland). Soy flour, spelt flour and oat bran, all from organic production, and spruce wood chips were purchased at a local supermarket (Coop, Lausanne, Switzerland). Wheat straw flour was purchased from Provimi Kilba (Cossonay, Switzerland). Dry rushes (*Juncus* genus, stem diameter: 0.2-0.4 mm), dry ash branches (*Fraxinus* genus, with bark, diameter of the branches: 0.3-0.7 mm) and dry beech sawdust (*Fagus* genus) were collected in a wetland and in the forest next to L’Isle (Switzerland). Oat bran and spruce wood chips were ground to obtain fine particles (< 1 mm). Ash branches and rushes were cut into sections of 0.5-1.0 cm, washed with tap water and oven-dried for 24 h at 60°C.

### Microorganisms and inoculum preparation

Pure strains of *S. cyaneus* CECT 3335 and *S. ipomoea* CECT 3341 (from Spanish Type Culture Collection, Valencia, Spain), *S. griseus* NBRC 13350 (from NITE Biological Resource Center, Chiba, Japan) and *S. psammoticus* MTCC 7334 (from Microbial Type Culture Collection, Chandigarh, India) were cultivated in GYM *streptomyces* medium (DSMZ, medium 65 (in g l^-1^): glucose – 4, yeast extract – 4, malt extract – 10, pH 7.2) at 30°C, 140 rpm during 4 d. Cell pellets were collected by centrifugation, washed 3 times with phosphate-buffered saline (PBS (in g l^-1^): NaCl – 8, KCl – 0.2, Na_2_HPO_4_ – 1.44, KH_2_PO_4_ – 0.24, pH 7.4) and then stored as cells suspension (with typical cell density of ~7 × 10^3^ CFU ml^-1^) in PBS with 5% glycerol at −80°C to be used as inoculum. The strain *T. versicolor* ATCC 42530 (from American Type Culture Collection, Manassas, Virginia, USA) was maintained by sub-culturing it every 30 d on 20 g l^-1^ malt extract agar (15 g l^-1^) slants (pH 4.5) at 25°C. A mycelial suspension of *T. versicolor* was prepared by homogenizing 5–7 d grown mycelium in malt extract medium (20 g l^-1^, pH 4.5) as described by Blánquez et al. (2004), and then stored in saline solution (NaCl – 8 g l^-1^) at 4°C until use as inoculums (8.5 g l^-1^ dry volatile solid mycelium).

### Laccase production

Production of laccase by the four *Streptomyces* strains was done in ISP9 mineral medium (Shirling & Gottlieb 1966) composed of (in g l^-1^): (NH_4_)_2_SO_4_ – 2.64, KH_2_PO_4_ anhydrous – 2.38, K_2_HPO_4_ · 3H_2_O – 5.65, MgSO_4_ · 7H_2_O – 0.1, with the following trace elements (in mg l^-1^): FeSO_4_ · 7H_2_O – 1.1, ZnSO_4_ · 7H_2_O – 1.5, CuSO_4_ · 5H_2_O – 6.4 and MnCl_2_ · 4H_2_O – 7.9, pH 6.6 – 6.9. In this mineral medium, five different carbon sources were tested at 10 g l^-1^: soy flour, oat bran, glucose, wheat straw flour and spruce sawdust. Production of laccase activity by *S. cyaneus* was also tested in a modified and optimized ISP9 mineral medium, with 6.4 times less copper (1 mg l^-1^ CuSO_4_ · 5H_2_O), and with the same five different carbon sources (at 10 g l^-1^) except glucose, which was replaced by spelt flour.

Finally, to test the ability of *S. cyaneus* and *T. versicolor* to produce laccase activity in wastewater, secondary treated wastewater was collected (grab sample) at the Lausanne (Switzerland) municipal WWTP in the effluent of a moving bed bioreactor with full nitrification. The ionic wastewater composition, measured by ion chromatography–conductivity detector (Dionex DX 500), was (in mg l^-1^): P-PO_4_^2-^ – 1.0, SO_4_^2-^ – 229, Cl^-^ – 837, N-NO_3_^-^ – 93, N-NH_4_^+^ – 0.09, Mg^2+^ – 11.5, Ca^2+^ – 83, Na^+^ – 74, K^+^ – 15.4. In this wastewater, five different sources of carbon were tested: soy flour (10 g l^-1^, initial pH after substrate addition: 6.8), spelt flour (10 g l^-1^, pH 7.1), rushes (20 g l^-1^, pH 5.5), ash branches (100 g l^-1^, pH 4.8) and beech sawdust (20 g l^-1^, pH 5.9).

The liquid media and the wastewater, together with their carbon sources, were autoclaved 30 min at 121°C and then inoculated with 0.33% or 0.67% (v/v) of, respectively, *Streptomyces* and *T. versicolor* inocula. Cultures were incubated at 30°C for 23 d and shaken at 140 rpm to ensure aerobic conditions. Every 1–3 d, 1.5 ml was withdrawn in aseptic conditions from each culture flask and centrifuged at 10,000 *g* for 20 min. Cell-free culture supernatants were then used directly to determine laccase activity and pH. At the end of the incubation period, cell-free supernatants from the remaining cultures showing the highest activity were collected with the same procedure, filtered at 0.45 μm (Filtropur, Sarstedt), and stored at −20°C until they were used as extracellular crude enzyme preparations.

### Concentrated *S. cyaneus* laccase preparation

For micropollutant degradation and laccase stability assays, as laccase activity in the extracellular crude enzyme preparation was not always high enough, 100 ml of cell-free culture supernatant of *S. cyaneus* (in modified ISP9 medium with soy flour), filtered at 0.45 μm, were concentrated 33 times by ultrafiltration (Vivaspin 20 centrifugation devices, PES membranes, MWCO: 30 kDa, from Startorius AG, Göttingen, Germany) to obtain 3 ml of laccase concentrated at ~2000 U l^-1^.

### Laccase activity test

Laccase activity was determined using a colorimetric assay by measuring the oxidation of 0.5 mM ABTS in oxygen-saturated acetate buffer (0.1 M) at pH 4.5 and 25°C as described by Margot et al. (2013b). Crude laccase preparation was added to the solution and the increase of absorbance at 420 nm was monitored with a temperature-controlled spectrophotometer (U-3010, Hitachi, Tokyo, Japan). One unit of activity (U) was defined by the oxidation of one μmol of ABTS per min, using the extinction coefficient ϵ_420nm_ of 36,000 M^-1^ cm^-1^ (Childs & Bardsley 1975).

The laccase ability to oxidize other substrates was determined by the same procedure, monitoring the oxidation at 468 nm (ϵ_468nm_: 27,500 M^-1^ cm^-1^) (Muñoz et al. 1997), 470 nm (ϵ_470nm_: 26,600 M^-1^ cm^-1^) (Koduri & Tien 1995) and 526 nm (ϵ_526nm_: 65,000 M^-1^ cm^-1^) (Palmieri et al. 1997) for, respectively, 2,6-dimethoxyphenol (DMP, at 0.5 mM), guaiacol (at 0.5 mM) and syringaldazine (at 0.01 mM, stock solution of 0.216 mM in methanol).

### Influence of the pH on laccase activity

Laccase activity was measured at different pH values, from 2.6 to 8, in citric acid (2 – 40 mM) - dibasic sodium phosphate (8 – 130 mM) buffers, with four different substrates: ABTS (0.5 mM), DMP (0.5 mM), syringaldazine (0.01 mM) and guaiacol (0.5 mM). Aliquots of 200 μl of *S. cyaneus* crude laccase preparation (L_*Sc*_), or 30 to 200 μl of *T. versicolor* commercial laccase solution (L_*Tv*_, 0.1 g l^-1^) were added to a total of 1200 μl of reaction mixture, the activity of which was measured at 25°C as described above. The pH was measured in the solution after addition of the laccase preparation. Measurements were conducted in duplicate.

### Temperature influence on the activity

Laccase activity was measured at different temperatures, from 10 to 80°C, in acetate buffer (0.1 M, pH 4.5 at 25°C), with 0.5 mM ABTS. Aliquots of 30 to 200 μl of *S. cyaneus* crude laccase preparation, or 30 μl of *T. versicolor* commercial laccase solution (0.1 g l^-1^) were added to 1200 μl of reaction mixture after which the activity was measured as described above. The temperature and pH were checked in the spectrophotometer cuvettes before and after the reaction. Measurements were conducted on 2 to 3 replicates. As the pH of the acetate buffer decreased when the temperature increased (Additional file 1: Figure S1, Supporting information (SI)), the measured activities were corrected to an equivalent activity at pH 4.5, as described in the SI, section 1.

### Stability at different pH

Laccase stability was assessed in pure water, as well as in buffer solutions at different pH. Citric acid (5 – 20 mM) - dibasic sodium phosphate (10 – 40 mM) buffers were used for pH 3 to 7, and Tris – HCl buffers (50 mM) for pH 8 and 9. Concentrated *S. cyaneus* crude laccase preparation or commercial *T. versicolor* laccase were added to the buffers to reach an initial laccase activity of 130 U l^-1^ and then incubated in the dark at 25°C for 55 d. The laccase activity and the pH in the solutions were monitored regularly. Experiments were conducted in duplicate.

### Inhibition by sodium chloride

The inhibitory effect of sodium chloride was assessed by measuring the laccase activity with ABTS in acetate buffer (0.1 M, pH 4.5) containing from 0 to 600 mM (0–35 g l^-1^) of NaCl. Crude *S. cyaneus* laccase preparation or commercial *T. versicolor* laccase were added to the solution (initial laccase activity without inhibitors of 10 U l^-1^), incubated for 30 s, before measuring the activity with the addition of ABTS (0.5 mM).

### Micropollutant analysis

Determination of BPA, DFC and MFA concentrations was carried out by reverse phase liquid chromatography with a diode-array detector (HPLC-DAD) (LC-2000plus, Jasco, Tokyo, Japan, equipped with Bondapack-C18 column, 15–20 μm, 3.9 mm × 300 mm, Waters^TM^, Milford, USA) as described by Margot et al. (2013b). Briefly, separation of the compounds was conducted with a 20-min gradient, at 1 ml min^-1^, of pure H_2_O containing 0.1% acetic acid and increasing concentration of methanol from 40 to 65% (v/v). Detection of the compounds was done by DAD at 200, 224 and 278 nm. The limit of detection (LOD) was around 0.3 mg l^-1^ (~1 μM).

### Micropollutant oxidation assay with laccase at different pH

Micropollutant oxidation assays were performed as previously described by Margot et al. (2013b) in citrate phosphate buffer (30–40 mM) at three different pH values (5, 6 and 7) with a mixture of the three compounds at 20 mg l^-1^ each: DFC, MFA and BPA. Relatively high concentrations were tested to use a fast and simple analytical method (HPLC-DAD). As the pollutant stock solutions were prepared in methanol or acetone, the reaction mixture contained at the end 4% (v/v) of methanol and 2% of acetone, which did not significantly influence laccase activity. Batch reactions were conducted in 2-ml glass vials containing 1 ml of oxygen-saturated reaction mixture. Reactions were initiated by adding laccase preparation to obtain an initial activity of 210–220 U l^-1^. For *T. versicolor* laccase, a stock solution of commercial enzyme (1 g l^-1^ in pure water) was used. For *S. cyaneus* laccase, concentrated crude enzyme preparation (laccase activity of 2000 U l^-1^) was added. The vials were incubated in the dark at 25°C under static conditions for 12 d. As shown in a previous study (Margot et al. 2013b), diffusion of oxygen from the air space was sufficient to maintain a high level of dissolved oxygen during the reaction. After defined reaction times, aliquots (50 μl) were withdrawn from each vial and directly injected into the HPLC column to analyse micropollutant concentrations. Controls without laccase were performed at the three pH values to assess chemical degradation. Duplicate experiments were conducted. Laccase activity and pH were analysed at the beginning and at the end of the incubation period in each vial. The pH stayed stable during the experiments in all the vials.

## Results

### Production of laccase activity by *Streptomyces* strains

Among the four strains of *Streptomyces* tested in ISP9 medium, laccase activity was only detected in the culture supernatant of *S. cyaneus* (with soy flour: 35 U l^-1^, oat bran: 2.75 U l^-1^ and glucose: 3.75 U l^-1^) and *S. ipomoea* (with soy flour: 0.75 U l^-1^ and oat bran: 0.5 U l^-1^), despite notable growth of all four strains in the media containing soy flour and oat bran. No laccase activity was detected in the cultures of *S. psammoticus* and *S. griseus*, neither in ISP9 medium with the five different carbon sources, nor in another specific medium with wheat straw and yeast extract, as described by Niladevi and Prema (2008). The absence of activity with *S. psammoticus* strain MTCC 7334 contrasts with studies of Niladevi et al. (2008). Although *S. griseus* was reported to produce extracellular laccase, this enzyme is assumed to be mainly localized in the cell wall (Endo et al. 2002), which could explain the absence of activity detected in the culture supernatant. No activity was detected in any culture when wheat straw flour and spruce sawdust were used as the sole carbon source. Depending on the substrate, *S. cyaneus* produced from 5.5- to 46-times more laccase activity than *S. ipomoea*, making this strain the best candidate among the tested *Streptomyces* strains for laccase production during wastewater treatment. Thus, only *S. cyaneus* was selected for further characterization.

**Figure 1:**
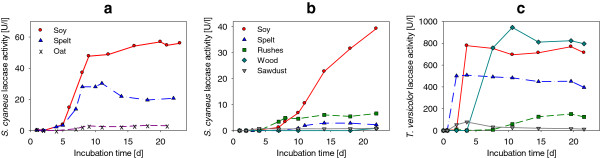
**Laccase activity in the culture supernatants. (a)***S. cyaneus* cultivated in modified ISP9 medium with soy flour, spelt flour and oat bran as carbon sources, **(b)***S. cyaneus* cultivated in secondary treated sterile municipal wastewater with soy flour, spelt flour, rushes pieces, ash branches pieces (wood) and beech sawdust, and **(c)***T. versicolor* cultivated in treated wastewater with the same carbon sources.

Laccase activity in the supernatant of *S. cyaneus* cultures was enhanced in modified ISP9 medium (containing 6.4 times less copper) (Figure 1a), reaching on average 57 U l^-1^ with soy flour and 30 U l^-1^ with spelt flour. Similar activities (200 U l^-1^ at 50°C, equivalent to about 50 U l^-1^ at 25°C) were measured with the same strain after 14 d of growth with soy flour by Moya et al. (2010). The activity increased rapidly after 4–5 d of incubation, once the strain had reached the stationary phase (Additional file 1: Figure S2, SI). Similar observations were made by Arias et al. (2003), who suggested that this increase in activity was related to cell death and lysis releasing intracellular laccase. After 8–9 d of incubation, laccase production decreased and the activity reached a plateau, staying at a similar level until the end of the incubation (23 d).

### Laccase production in treated wastewater

Both *S. cyaneus* and *T. versicolor* were able to grow in sterile secondary treated wastewater containing different carbon sources. In *S. cyaneus* culture supernatant (Figure 1b), laccase activity was observed with soy flour (with a similar level to that in ISP9 medium but delayed by 2 weeks) and spelt flour (10 times lower than in ISP9), but also with rushes (6.6 U l^-1^), suggesting that lignocellulose-containing waste could serve as substrate for laccase production. However, no or only very low activity levels (< 1 U l^-1^) were observed with wood branches or sawdust, possibly due to the low pH (4.7) present in the wood medium and probable lack of essential nutrients (nitrogen and phosphorus) with sawdust as the sole substrate.

Laccase activity of *T. versicolor* cultures in wastewater increased very rapidly after only 2–3 d of incubation (Figure 1c), reaching a maximum of 508, 778 and 945 U l^-1^ for spelt flour, soy flour and wood branches, respectively. Lower activity was observed with rushes (151 U l^-1^) and sawdust (79 U l^-1^) but, unlike *S. cyaneus*, all lignocellulose substrates led to the presence of laccase activity in culture supernatant. High activity (e.g., 550 U l^-1^ with wood branches and soy flour) was still measured after 45 d of incubation (data not shown), showing the ability of this fungus to survive in the long term on these lignocellulosic substrates. Laccase activity was 20-times higher in *T. versicolor* culture supernatant with soy flour and rushes than for *S. cyaneus*, and 175-times higher with spelt flour. Wood branches were the best substrate for *T. versicolor* laccase production.

*T. versicolor* produces two main laccase isoenzymes, the proportions of which differ depending on the growth substrate or the presence of inductors (Bourbonnais et al. 1995; Moldes et al. 2004; Nakatani et al. 2010). As the kinetic properties of these two main isoenzymes differ slightly (Bourbonnais et al. 1995; Moldes & Sanromán 2006), different proportions of isoenzymes in the mixture can lead to slightly different oxidation behavior. The commercially available preparation which is a mixture of different proteins with at least two distinct enzymes displaying laccase activity (Additional file 1: Figure S3, SI) had very similar micropollutant oxidation activity as the laccase produced by *T. versicolor* on wood substrate (Additional file 1: Figure S4, SI). Thus, to allow comparison with literature data and to have a reproducible and constant proportion of the different laccase isoenzymes, commercial *T. versicolor* laccase preparation was used for the following experiments instead of culture supernatants.

### Influence of pH on laccase activity with different substrates

**Figure 2:**
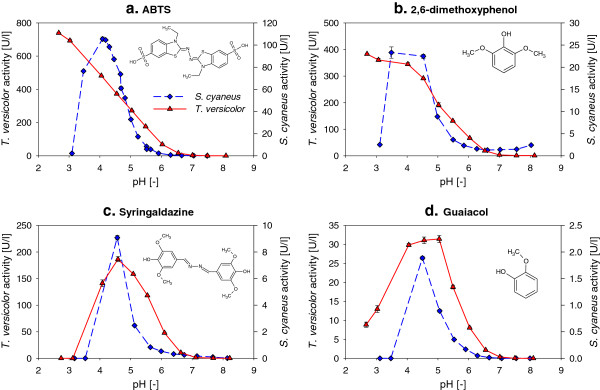
**Influence of pH on *S. cyaneus *(♦) and *T. versicolor *(▲) laccase activity with different substrates: (a) ABTS, (b) 2,6-dimethoxyphenol, (c) syringaldazine and (d) guaiacol.** Average and values of duplicates, at 25°C.

As shown in Figure 2, laccase preparations of *S. cyaneus* (L_*Sc*_, from the culture supernatants) and commercial laccase of *T. versicolor* (L_*Tv*_) were both able to oxidize the four substrates tested, as also observed in other studies (Arias et al. 2003; Eichlerová et al. 2012). Compared to its activity with ABTS at pH 4.5 (close to the optimum), L_*Sc*_ was 4-, 10- and 46-times less active with DMP, syringaldazine and guaiacol, respectively. L_*Tv*_ was only 1.3-, 2- and 12-times less active with these three substrates compared to ABTS, showing a broader substrate specifity than L_*Sc*_.

For the four laccase substrates, the pH had a very strong influence on the activity of both laccase preparations, with very low activity in slightly alkaline conditions (pH > 7) and maximum activity between pH 4 and 4.5 for L_*Sc*_, and from less than 2.7 to 5 for L_*Tv*_ (Figure 2). L_*Sc*_ activity was strongly dependent on the pH, with, for instance, an order of magnitude increase between pH 5.5 and 4.5 with ABTS (from 8.4 to 87 U l^-1^) and with syringaldazine (from 0.8 to 9 U l^-1^), and a rapid decrease of activity below pH 3.5 with all substrates.

### Influence of temperature on laccase activity

**Figure 3:**
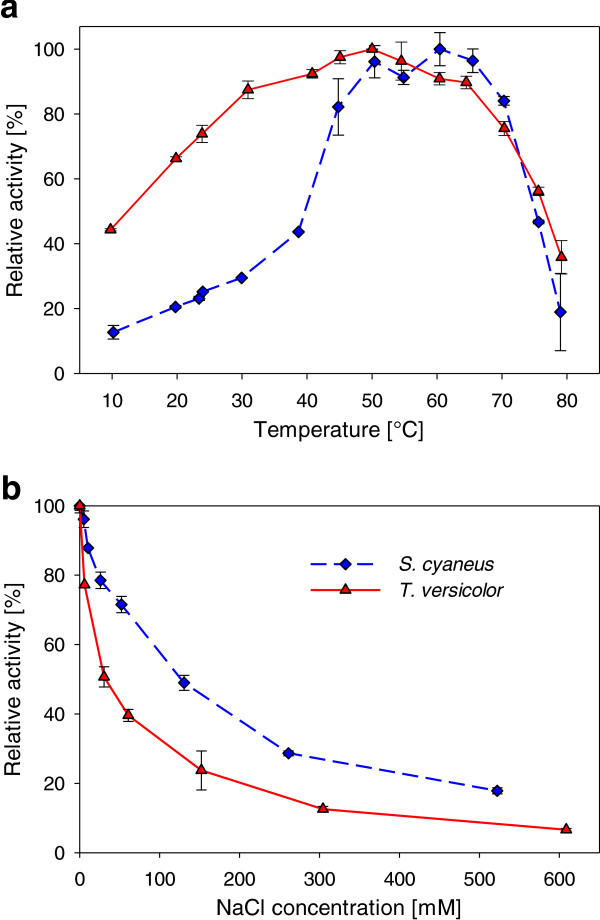
**Influence of temperature (a) and sodium chloride concentration (b) on *S. cyaneus *(♦) and *T. versicolor *(▲) laccase activity.** Average ± standard deviation of 2 to 3 replicates, at pH 4.5.Inhibition experiments with NaCl were performed at 25°C, with an initial laccase activity (without NaCl) of 10 U l^-1^.

Maximum activities (with ABTS, pH 4.5) were observed at 60°C and 50°C for L_*Sc*_ and L_*Tv*_, respectively (Figure 3a), which is 10°C lower than optimal temperatures reported in other studies (70°C and 60°C respectively) (Arias et al. 2003; Rancaño et al. 2003). A rapid decrease in activity was observed above 70°C for both preparations, probably due to heat denaturation of the enzymes. For L_*Sc*_, a rapid decrease in activity was also observed when the temperature decreased below 50°C, with only 25% of its maximum activity remaining at 25°C, compared to 73% for L_*Tv*_. Both laccase preparations were still active at 10°C, showing 13 and 44% of their maximum activity for L_*Sc*_ and L_*Tv*_, respectively.

### Inhibition of the laccase activity by NaCl

Both laccase preparations were sensitive to sodium chloride (Figure 3b), with, for L_*Sc*_ and L_*Tv*_ respectively, 4 and 20% of activity inhibition at 5 mM, a typical concentration for municipal wastewater, and more than 80 and 90% at 550 mM, a concentration found in various industrial wastewaters and in seawater (Lefebvre & Moletta 2006; Leutz 1974). The IC_50_ (inhibition concentration for which the activity was reduced by 50%) was observed at 130 mM for L_*Sc*_ and at 30 mM for L_*Tv*_, showing the higher sensitivity of the latter towards chloride ions. Similar IC_50_ (around 20 mM Cl^-^) were observed for L_*Tv*_ by Enaud et al. (2011), but no information on L_*Sc*_ chloride inhibition was reported previously.

### Stability of laccase at different pH

**Figure 4:**
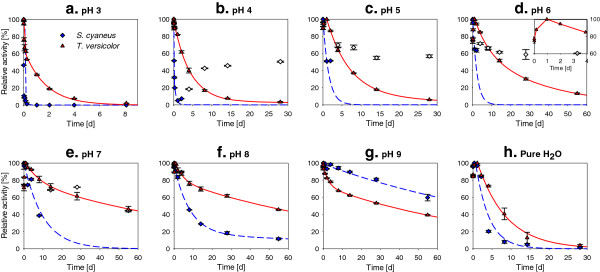
***S. cyaneus *(♦) and *T. versicolor *(▲) laccase residual activity as a function of incubation time at different pH in buffer solution at 25°C.** Plots show the average and values of duplicates. Relative activity refers to the maximum activity measured during the test. The initial laccase activity was 130 U l^-1^ in all the tests. Lines: bi-exponential inactivation model fitted to the data. The white symbols (◊) represent *S. cyaneus* laccase residual activity in the tests where the pH increased gradually to 7.3-7.7 due to microbial growth. Due to change in pH, these data were not used to fit the model. The insert at pH 6 shows the increase of *T. versicolor* laccase activity during the first days of incubation, also observed at pH 5, 7 and 8.

The stability of laccase incubated at different pH and 25°C is presented in Figure 4. The data were fitted with a bi-exponential equation to model various mechanisms of enzyme inactivation (Aymard & Belarbi 2000), as described in the SI, section 2. The results of the fitting and the estimated half-life of laccase at different pH are presented in Additional file 1: Table S1 (SI). In most cases, a fast initial inactivation rate, represented by a high apparent first-order rate constant (*k*_2_), followed by slower decay kinetics (*k*_1_) were observed.

L_*Tv*_ was more stable than L_*Sc*_ in acidic and neutral pH. Very fast inactivation of L_*Sc*_ was observed at pH 3, with a half-life shorter than 3 min compared to 11 h for L_*Tv*_. At this pH, instant precipitation appeared when L_*Sc*_ was added. At pH 4, no precipitate was visible, but L_*Sc*_ was still rapidly inactivated, with a half-life of 90 min. L_*Sc*_ and L_*Tv*_ stability increased as the pH increased, reaching the highest stability at pH 9 for L_*Sc*_, with an estimated half-life of 82 d, and at pH 7–8 for L_*Tv*_, with a half-life of 47 d. When laccase was incubated in pure water (pH 6.5-7.5), the stability was significantly reduced compared to storage in a buffer at neutral pH; with half-life of only 3 and 6 d for L_*Sc*_ and L_*Tv*_, respectively.

From pH 5 to 8, an increase in L_*Tv*_ activity was observed during the first 24 h of incubation, as illustrated for pH 6 in Figure 4d. At pH 6 and 7, this increase in activity was as high as 34%. A similar increase in activity (24 ± 1%) was also observed after sonication (15 pulses of 3 s at 100 W) of fresh laccase solutions, suggesting that this increase was due to a better dispersion of the enzymes that were initially partly aggregated (presence of particles with strong laccase activity). This phenomenon was also noticed in other studies (Margot et al. 2013b; Silvério et al. 2013). Changes of the storage conditions (pH and temperature) could also gradually influence laccase activity, possibly due to slow reorganisation of laccase structure or conformation (Kurniawati & Nicell 2008).

**Figure 5:**
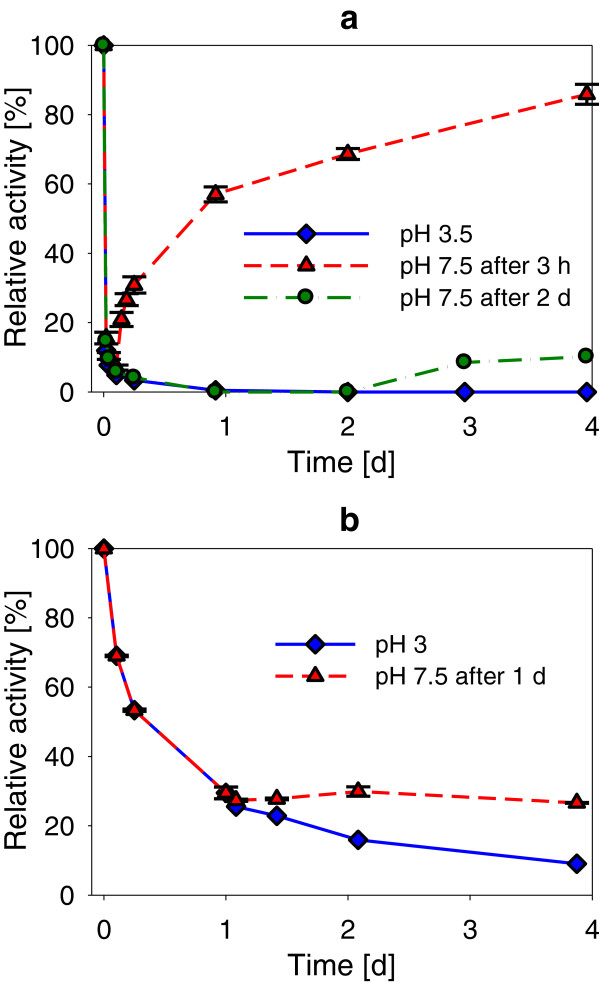
***S. cyaneus *(a) and *T. versicolor *(b) laccase residual activity as a function of incubation time at pH 3-3.5 (♦), and reversible inactivation due to alkalinisation at pH 7.5.** Alkalinization (NaOH addition) was done after 3 h (▲) or 2 d (●) for *S. cyaneus* laccase, and after 1 d (▲) for *T. versicolor* laccase. Average and values (error bars) of duplicates, incubated at 25°C in buffer solution.

As the tests were not conducted in sterile conditions, bacterial growth (turbidity, confirmed by microscopy) was observed after 2 d in the incubation tubes containing L_*Sc*_ at pH 4 to 7, resulting in an increase in the pH to 7.3-7.7 in all these tubes (Additional file 1: Figure S5, SI). An increase in activity following the increase in pH was observed (Figure 4b-e), suggesting a partially reversible pH inactivation of the enzyme. To verify this hypothesis, L_*Sc*_ and L_*Tv*_ were again incubated at pH 3.5 and 3, respectively, and their residual activity was followed over time. At a certain time, the pH of the solution of two of the four replicates was increased to 7.5 by addition of concentrated NaOH. The effect of this artificial increase in pH on laccase stability is presented in Figure 5. For L_*Sc*_, as expected, a very fast inactivation was observed in the four replicates, with more than 90% of inactivation within 1 h (Figure 5a). However, when the pH was increased to 7.5 after 3 h of incubation at pH 3.5, L_*Sc*_ activity increased again, reaching 86% of the initial activity after 4 d. At the same time, the precipitate observed at pH 3.5 was again solubilised at pH 7.5. In the replicates maintained at pH 3.5, the activity reduced to an undetectable level after 1 d. After 2 d at pH 3.5, pH was increased to 7.5 in one replicate, leading to a slow L_*Sc*_ activity increase from the no-detect level to 10% of the initial activity. These results indicate that L_*Sc*_ is affected by two inactivation types, one that is fast but reversible and another that is slower but irreversible. The former type is exactly what was observed in the stability experiment (depicted in Figure 4, b-e) when the pH increased due to bacterial activity. The irreversible L_*Sc*_ inactivation seemed to be relatively similar to L_*Tv*_ inactivation (Figure 4e), which seemed to be only affected by pH in an irreversible manner (Figure 5b).

### Micropollutant oxidation by laccase preparations

**Figure 6:**
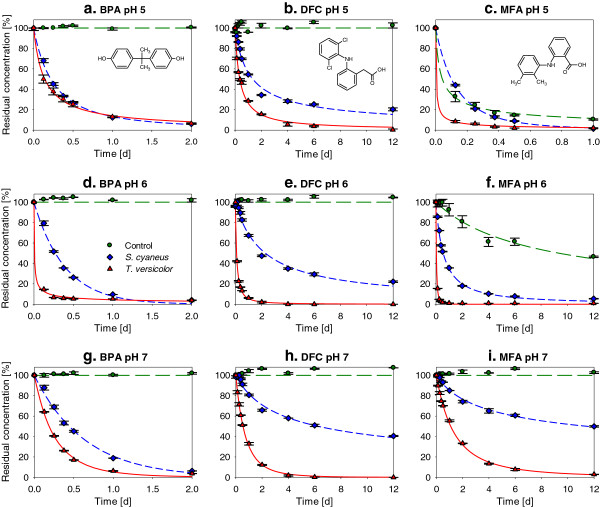
**Residual concentrations of three micropollutants as a function of the reaction time with laccase preparations from *S. cyaneus *(♦) and *T. versicolor *(▲), and in a control without laccase (●).** Degradation of bisphenol A (BPA), diclofenac (DFC) and mefenamic acid (MFA) at three different pH values (5, 6 and 7), at 25°C. Average and values of duplicates. The initial micropollutant concentration, present in mixture, was at 20 mg l^-1^ for the three compounds. The initial laccase activity was 210 and 220 U l^-1^ for *T. versicolor* and *S. cyaneus* laccases, respectively. Lines: variable order reaction model fitted to the data.

The kinetics of laccase-mediated degradation of the plastic additive BPA and the two anti-inflammatory drugs DFC and MFA at different pH are presented in Figure 6. The residual concentrations were fitted with a variable order reaction model (Margot et al. 2013b) as described in the section 3 of the SI. The results of the fitting and the estimated half-life of the pollutants at different pH are presented in Additional file 1: Table S2 (SI).

Both laccase preparations were able to oxidize the three pollutants at all pH values studied. Except for BPA at pH 5, where both laccases had a very similar efficiency, L_*Tv*_ provided more efficient micropollutant oxidation, especially at pH 6 and 7. As observed previously (Margot et al. 2013b), MFA was degraded in the control without laccase under acidic conditions, with a half-life of 1 h at pH 5 and 9 d at pH 6. Thus, at pH 5, it was difficult to distinguish between laccase oxidation and abiotic degradation. However, at pH 6 and 7, MFA was significantly oxidized by both laccases. The highest oxidation rates of BPA and DFC were observed at pH 6 for L_*Tv*_, with half-lives of 9 min and 2.2 h, respectively, and at pH 5 for L_*Sc*_ with half-lives of 5.2 h and 32 h, respectively.

The residual laccase activities in the reaction mixtures at the end of the test, after 12 d of incubation, are presented in Table 1. Loss of 97% of the initial activity was observed at pH 5 for L_*Tv*_. Additional laccase inactivation (from 0 to 17%) seemed to occur over the course of the reaction, especially at pH 5, compared to incubation in solutions without micropollutants (stability test). Unlike what was observed for oxidation of phenols (Kurniawati & Nicell 2008), the laccase inactivation due to the catalytic reaction was, however, much lower than the inactivation due the test conditions (pH and temperature). At the end of the test, L_*Tv*_ activity was 17-times higher in the mixture at pH 6 than at pH 5. The residual activity of L_*Sc*_ was slightly higher than that for L_*Tv*_. This contrasts with the results of the stability test, where L_*Sc*_ was rapidly, but reversibly, inactivated at these pH values.

**Table 1:** Residual laccase activity in the reaction vials after 12 d of reaction with the mixture of micropollutants at 25°C and at different pH

	**Residual activity**^**a**^		**Residual predicted activity**^**b**^
	*T. versicolor*	*S. cyaneus*	*T. versicolor*
	[%]	[%]	[%]
pH 5	3.3 (± 0.2)	10.4 (± 0.2)	20
pH 6	53.7 (± 0.9)	52.0 (± 2.1)	53
pH 7	68.4 (± 1.0)	79.1 (± 0.1)	74

^a^Average and standard deviation of duplicates, relative to the initial activity.

^b^Residual activity after 12 d predicted with the bi-exponential model based on the stability test (incubation at 25°C at the same pH but without micropollutants).

## Discussion

For selection of microorganisms able to produce laccase on-site for treating micropollutants in wastewater, the potential of four strains of *Streptomyces* bacteria together with the white-rot fungus *T. versicolor* was assessed for (i) their ability to produce laccase in treated wastewater on cheap substrates, (ii) their laccase activity at different pH and temperature, (iii) laccase inhibition by chloride salt, (iv) laccase stability, and (v) the laccase substrate range and their ability to oxidize different micropollutants.

### Laccase production on different substrates

Among the four *Streptomyces* strains assessed, only *S. cyaneus* produced laccase to a level potentially sufficient for the targeted application, for instance in a system with a long hydraulic residence time (of a few days to a few weeks such as wetlands). Despite the attractive possibility of producing (low amounts of) laccase in treated wastewater on lignocellulosic substrates (rushes) with *S. cyaneus* without the addition of other nutrients, the activity levels reached were not comparable with those obtained in *T. versicolor* cultures (which were more than 20-times higher). *T. versicolor* was found to produce high amounts of laccase (up to 945 U l^-1^) in treated wastewater with ash branches (including the bark) as the sole substrate, which is promising for the development of a fungal trickling filter for wastewater post-treatment. Indeed, this forestry waste is cheap and widely available in Switzerland, for example, and the activity reached in the supernatant is high enough – according to a previous study (Margot et al. 2013b) – to allow high removal (> 90%) of various micropollutants (BPA, DFC, MFA and triclosan) in an appropriate time range (less than 10 h) at pH 7 and 25°C, conditions that are found in municipal wastewaters. Maintaining *T. versicolor* in unsterile biologically treated wastewater is, however, still a challenge due to competition/predation by other microorganisms. This competition can probably be limited in case of use of lignocellulosic materials as the sole substrate, as only few organisms can use them as carbon source.

The composition of the growth substrate had a strong influence on laccase production by *T. versicolor*, as also observed in other studies on lignocellulosic materials (Özşölen et al. 2010). As only complex substrates were used here (wood with bark, integral soy flour, etc.), it was not possible to identify which components induced laccase production. It seemed that the role of wood bark was significant as much lower activity (79 U l^-1^) was observed in *T. versicolor* cultures with only beech sawdust compared to that with ash branches (with the bark). These results were also confirmed by an additional experiment in treated wastewater (Additional file 1: Figure S6, SI), where high *T. versicolor* laccase activity (> 550 U l^-1^) was observed in culture supernatant after 9 d of cultivation on poplar (*Populus* spp.) branches with the bark, on reed pieces (*Phragmites australis*) and on wheat straw, and low activity (< 30 U l^-1^) on pine wood chips (without bark) and glucose. Bark contains in general more lignin and polyphenols than wood (Harkin & Rowe 1971), and aromatic or phenolic compounds related to lignin or lignin derivatives such as ferulic acid or vanillin, are known to induce laccase production by white-rot fungi (De Souza et al. 2004; Parenti et al. 2013). For *S. cyaneus*, induction of laccase production on lignocellulosic substrate was not observed. The role of laccase in *Streptomyces* spp. is, however, not clear and might be related more to morphogenesis than to lignin degradation (Endo et al. 2002). This is also supported by the means by which *S. cyaneus* likely produces extracellular laccase (cell lysis rather than active secretion). Indeed, the *S. cyaneus* laccase sequence deposited by Moya and co-workers (GenBank HQ857207) does not harbor any secretion signal peptide (J. Maillard, unpublished data).

### Laccase activity at different pH values and temperatures

Both laccases had optimal activity under acidic conditions (pH < 5) for all substrates. Slight variations of the optimal pH (< 3 to 5) were observed for the phenolic substrates, which are assumed to be related to the protonation/deprotonation state of the compound (Rosado et al. 2012). The pH range in which significant activity was measured was wider for L_*Tv*_ than for L_*Sc*_, with L_*Tv*_ showing higher activity in the pH range 5.5 to 7. Both laccases showed, however, rather low activity under slightly alkaline conditions, impairing their use in non-acidified municipal wastewater (pH 7–8).

The strong pH influence observed on the activity of both laccases on all the substrates could be related to a balance between two opposing phenomena (Xu 1997): (i) the increase in redox potential difference (and thus oxidation rate) between laccase type 1 copper site (T1, where the substrate oxidation takes place) and the phenolic or aniline substrates when the pH increases, and (ii) the increase in hydroxyl inhibition of laccase (binding of the hydroxide anion to the T2/T3 Cu, where the reduction of oxygen to water take place) at higher pH, possibly leading to a bell-shape activity profile. Despite their relatively low amino acid sequence homology (Additional file 1: Figure S7, SI), similar mechanisms are expected for both laccases as the three-dimensional structure of the active site of *S. cyaneus* laccase is presumably very similar to the one of *T. versicolor* laccase (see sections 9 and 10 of the SI for details). This bell-shape profile was observed for both laccases on the phenolic substrates syringaldazine and guaiacol, but not on ABTS with L_*Tv*_, which is consistent with the fact that the redox potential of ABTS is not dependent on the pH in the range tested (no protons involved in the oxidation) (Xu 1997). Due to its phenolic structure, a bell-shape profile was expected for DMP, but not observed with L_*Tv*_. The increase of activity with the pH was, however, possibly out of the pH range studied (appearing at lower pH). The low L_*Sc*_ activity below pH 3.5 with all substrates was probably due to the fast inactivation of the enzyme at these pH values (50% inactivation in 2 min at pH 3) than due to thermodynamic and kinetic considerations (variation of redox potential).

Both laccases had optimal activity on ABTS at 50 to 60°C. These optimal temperatures can, however, differ depending on the substrate (Margot et al. 2013b; Yang et al. 2013). L_*Tv*_ showed significant activity in a wider temperature range than L_*Sc*_, especially at lower temperatures (from 10 to 40°C). It retained 44% of its maximum activity at 10°C, making this enzyme more attractive for municipal wastewater treatment (10-25°C).

### Laccase inhibition by chloride

Municipal and especially industrial wastewaters can contain relatively high chloride concentrations. Chloride (Cl^-^), similar to other halide anions (F^-^, Br^-^) or to the hydroxide anion (OH^-^), has been reported to either bind to the T2 Cu of laccase and to interrupt the internal electron transfer between T1 and T2/T3 active site (Xu 1996), and/or to bind near the T1 active site, blocking the access of the substrate to T1 Cu or inhibiting the electron transfer (Enaud et al. 2011). Both laccases considered here were inhibited by sodium chloride, with L_*Sc*_ being slightly more tolerant. The chloride concentration in municipal wastewater which is around 2.5 to 5 mM (unpublished data, Lausanne WWTP), is not expected to affect laccase activity significantly (< 20%). However, chloride inhibition can be an issue for the treatment of industrial effluents from, for example, the pharmaceutical industry (around 90 mM Cl^-^ (Rajkumar & Palanivelu 2004)), especially for L_*Tv*_ (> 60% inhibition).

### Laccase stability

For all biotechnological applications, good stability of the enzyme under the treatment conditions is required. Enzyme inactivation is influenced by many different factors, the pH being an important one due to its effect on the structures of proteins (influence on the balance of electrostatic and hydrogen bonds between the amino acids) (Sadana 1988). L_*Sc*_ incubated at 25°C in buffer solutions was relatively rapidly inactivated (t_1/2_: 0–2 d) at acidic pH (< 7) compared to L_*Tv*_. However, this fast inactivation was reversible and L_*Sc*_ could recover most of its activity when the pH was switched again to alkaline conditions. The mechanism of this reversible pH inactivation is unknown, but could be due to refolding of the tertiary structure of the enzyme when the pH increases (Kurniawati & Nicell 2008), or possibly resolubilization of precipitated laccase. The irreversible L_*Sc*_ pH inactivation seemed to be in the same range as that observed for L_*Tv*_. Thus, if the reversible pH inactivation can be avoided, both laccases would have relatively similar stability. This was observed during the micropollutant degradation test. Indeed, the 12 d stability of L_*Sc*_ at pH 5, 6 and 7 during this test was similar or even higher than that of L_*Tv*_, and much higher than what was observed for L_*Sc*_ during the stability test. This suggests that the different incubation conditions, such as the presence of micropollutants (laccase substrate) and solvents (4% methanol and 2% acetone), prevented the reversible inactivation, possibly by limiting L_*Sc*_ precipitation/aggregation or by increasing the stability due to pollutant binding to the active centre of the enzyme (Mai et al. 2000). Laccase stability is thus not only dependent on the pH but also on the composition of the solution. This was also confirmed by the much higher laccase stability in buffer solution at pH 7 than in pure water at the same pH.

Similar stability results for L_*Tv*_ were previously reported (Kurniawati & Nicell 2008; Mai et al. 2000). In most cases, a fast initial inactivation rate followed by slower decay kinetics was observed. Some authors explained this behavior by the possible presence of two isoenzymes of laccase, one being unstable and rapidly inactivated and the other being more stable (Kurniawati & Nicell 2008). However, other mechanisms may also explain this biphasic behavior (Aymard & Belarbi 2000), such as a fast reversible inactivation followed by a slower irreversible one. *T. versicolor* is known to produce at least four different isoenzymes, which can differ significantly in their stability (Koschorreck et al. 2008). The commercially available *T. versicolor* laccase preparation used in this study contains, as shown in Additional file 1: Figure S3, SI, at least two distinct enzymes with laccase activity, possibly explaining the observed biphasic behavior. As the proportion of the different isoenzymes is reported to be influenced by the culture conditions (Moldes et al. 2004), stability may differ for other sources of *T. versicolor* laccase. The higher and relatively good laccase stability observed at neutral to alkaline pH values for both laccase preparations, L_*Tv*_ and L_*Sc*_, is advantageous for the targeted applications in municipal wastewater (pH 7–8). Although laccases are known to be relatively stable at ambient temperatures and near-neutral pH, this is, however, the first time that long-term stability (45-60% remaining activity after incubation 55 d at 25°C) was reported for these two particular laccase preparations.

### Laccase substrate range and oxidation of micropollutants

The broader the laccase substrate range is, the greater the potential for the enzyme to be used to remove micropollutants. Both laccases were able to oxidize the four aromatic model substrates tested, showing higher activity against the non-phenolic ABTS, followed by the phenolic compounds DMP, syringladazine and finally guaiacol. L_*Sc*_ was much less active on the phenolic substrates than on ABTS compared to L_*Tv*_. These differences in reactivity are reported to be related to differences in shape and chemical composition of the substrate binding site of the enzymes (Rosado et al. 2012; Xu et al. 1996). Differences in the phenol substitution seemed also to influence the activity. The electron-donating property of the methoxy group is reported to reduce the redox potential of phenolic compounds, guaiacol (1 methoxy group) having a higher redox potential *E*^0^ than the two other substrates (2 methoxy groups) (Xu 1996). For small *o*-substituted phenols, the redox potential difference (∆*E*^0^) between laccase type 1 copper site (T1) and the substrate seems to be the main driving force for the oxidation (Xu 1996). Therefore, the lower the *E*^0^ value of the phenolic substrate, the faster will be the reaction rate, which is consistent with the results obtained here. For larger *o*-substituents, other significant mechanisms such as steric hindrance may be observed (Xu 1996).

BPA, DFC and MFA are three common micropollutants found at relatively high concentrations in municipal WWTP effluent (average between 300–1000 ng l^-1^) (Kase et al. 2011). DFC is of special concern because it is not removed in conventional biological treatments (Margot et al. 2013a) and can affect fish at typical WWTP effluent concentrations (1 μg l^-1^) (Triebskorn et al. 2004). Despite their very low activity at pH 7 on the model substrates, both laccases were able to reduce the concentration of these micropollutants significantly at neutral pH, which is for the first time reported for bacterial laccases. The oxidation rates were much higher at pH 7 with L_*Tv*_ compared to L_*Sc*_, especially for the two aniline pollutants (DFC and MFA), confirming the wider pH range of this enzyme. L_*Sc*_ was less reactive with aniline (DFC and MFA) than with phenol compounds (BPA) compared to L_*Tv*_. Both laccases rapidly oxidized BPA, with a similar rate at pH 5, while L_*Tv*_ was more effective for the oxidation of the two aniline compounds at all pH values. This difference in oxidation rate is thought to be either related to different affinity for the aniline substrates or to a lower redox potential of the T1 copper site of L_*Sc*_, as observed for many other bacterial laccases (*E*^0^ < 0.5 V vs. SHE, compared to 0.785 V for L_*Tv*_) (Hong et al. 2011; Telke et al. 2009).

The higher DFC degradation with L_*Tv*_ at pH 6 than at pH 5 contrasts with a previous study where the highest removal was observed below pH 5 (Margot et al. 2013b). This shift in the optimal pH is likely due to the different initial enzyme concentrations used, 3.5 times higher in the previous study. At low enzyme concentrations, the oxidation rate was slower and the time to reach a defined level of micropollutant removal longer. The longer the reaction time, the higher was the loss of laccase by inactivation, especially under acidic conditions. Thus, in case of low enzyme concentration, the gain associated with higher laccase activity at lower pH was offset by the loss of activity at these pH values due to the long reaction time. Higher degradation levels were thus observed at higher pH, where laccase was more stable. The loss of activity was limited in case of high enzyme concentrations (fast reaction) and thus higher degradation levels were obtained at a lower pH value (close to the optimal pH for laccase activity). Therefore, depending on the laccase concentration, a compromise between laccase stability (higher at high pH values) and laccase activity (higher at low pH values) has to be found to determine the optimal pH for the treatment.

The evaluation of five laccase-producing organisms to improve micropollutant degradation in wastewater showed that *T. versicolor* was the most promising strain. This fungus produced more than 20-times more laccase activity than *S. cyaneus*, the best candidate of the *Streptomyces* strains evaluated, and this especially in treated wastewater with forestry waste as the sole substrate, a cheap and widely available product. Laccase from *T. versicolor* (L_*Tv*_) was moreover more active than that from *S. cyaneus* (L_*Sc*_) near neutral pH and between 10 to 25°C, conditions usually found in municipal wastewater. Despite an optimal activity under acidic conditions (pH < 6), which limits their use in non-acidified wastewater, both laccases had the ability to degrade common wastewater micropollutants, BPA, DFC and MFA even at neutral pH, which is for the first time reported for a bacterial laccase. Micropollutant oxidation was faster with L_*Tv*_, especially for aniline pollutants, showing the greater potential of this enzyme for the target application. Both laccases were relatively stable at slightly alkaline pH values, conditions found in municipal wastewater. Thus, altogether, despite a slightly lower resistance of its laccase to chloride, *T. versicolor* appeared to be the best candidate to be used in a post-treatment, such as a fungal trickling filter composed of wood support, for micropollutant degradation in wastewater.

## Competing interests

The authors declare that they have no competing interests.

## Supplementary Material

Additional file 1 Supplementary data associated with this article can be found in the online version. Click here for file
