# Supplementary material for: Bacterial versus fungal laccase: potential for micropollutant degradation
Source: AMB Express. 2013 Oct 24;3:63. doi: 10.1186/2191-0855-3-63 (PMC3819643; doi:10.1186/2191-0855-3-63)
Supplement: Additional file 1 — Supplementary data associated with this article can be found in the online version. [file 2191-0855-3-63-S1.pdf]

Supporting information

Bacterial *versus* fungal laccase: Potential for micropollutant degradation

Jonas Margot[1,2], Chloé Bennati-Granier[1,#], Julien Maillard[1], Paqui Blánquez[3], D. A. Barry[2], Christof Holliger[1]

[1] Laboratory for Environmental Biotechnology, School of Architecture, Civil and Environmental Engineering (ENAC), Station 6, Ecole Polytechnique Fédérale de Lausanne (EPFL), 1015 Lausanne, Switzerland

[2] Ecological Engineering Laboratory, School of Architecture, Civil and Environmental Engineering (ENAC), Station 2, Ecole Polytechnique Fédérale de Lausanne (EPFL), 1015 Lausanne, Switzerland

[3]Departament d'Enginyeria Química, Escola d'Enginyeria, Universitat Autònoma de Barcelona (UAB), 08193Bellaterra, Spain

[#] Present address: Unité de Biotechnologie des Champignons Filamenteux, Institut National de le Recherche Agronomique (INRA)/Universités de Provence et de la Méditerranée, Ecole Supérieure des Ingénieurs de Luminy, 163 avenue de Luminy-CP 925, 13288 Marseille Cedex 09, France

Corresponding author: Jonas Margot, jonas.margot@epfl.ch, Phone: +41-21-6938086, Fax: +41-21-6938035, Address: EPFL ENAC IIE ECOL, Station 2, 1015 Lausanne, Switzerland

Table of contents

1. Influence of the temperature on the pH - Correction of the activity to pH 4.5 (Fig. S1) 2

2. Fitting of a bi-exponential model to laccase activity stability (Table S1) 3

3. Fitting of a variable order reaction model to the oxidation of micropollutants (Table S2) 4

4. Evolution of extracellular laccase activity and biomass in *S. cyaneus* cultures (Fig. S2) 5

5. Characterization of the commercial laccase preparation from *Trametes versicolor* from Sigma (Fig. S3) 6

6. Comparison of "commercial" versus "in house-produced" *Trametes versicolor* laccases (Fig. S4) 7

7. Evolution of pH during the laccase stability test - example for the incubation at an initial pH of 4 (Fig. S5) 9

8. Production of laccase activity in treated wastewater by *T. versicolor* (Fig. S6) 10

9. Identification of one laccase candidate from *S. cyaneus* culture supernatant 11

10. Comparison of sequences of *S. cyaneus* laccase and *S. antibioticus* phenoxazinone synthase 12

11. Phylogenetic tree between different multicopper oxidases (Fig. S7) 13

12. References 14

Materials and methods – complementary information

1. Influence of the temperature on the pH of the acetate buffer - Correction of the activity to pH 4.5

During the test to assess the influence of the temperature on laccase activity, the pH of the acetate buffer in the cuvettes decreased when the temperature increased, from pH 4.62 at 10°C to pH 4.05 at 70°C, following a linear relation (valid between T = 2 and 70°C, R^2: 0.993): pH = -0.0099T (°C) + 4.715 (Fig. S1 a).

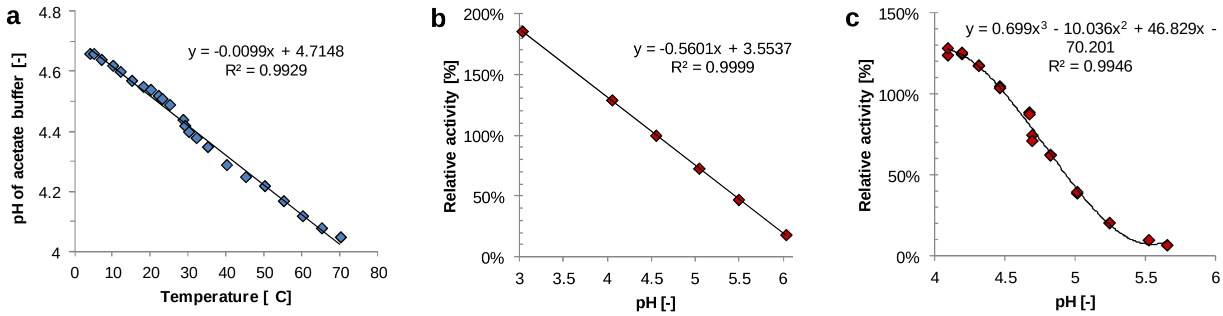

Fig. S1 a. Influence of temperature on the pH of 100-mM acetate buffer (pH 4.5 at 25°C). b. Influence of the pH on the ABTS activity of *T. versicolor* laccase. c. Influence of the pH on the ABTS activity of *S. cyaneus* laccase. Activities are given relative to that at pH 4.5 (set at 100%)

The laccase activity with ABTS increased when the pH decreased from 5 to 4 (Fig. S1 b and c). Therefore, to assess the temperature effect alone without the pH effect, the measured activity values (A_{pH}) were corrected to an equivalent activity at pH 4.5 ($A_{4.5}$) with the following relation: $A_{4.5} = f_{4.5}\ A_{pH}$. The correction factors $f_{4.5}$, determined by regression, were, for *T. versicolor* laccase (valid from pH 3 to 6, R^2: 0.999): $f_{4.5} = -0.5601\mathrm{pH} + 3.5537$, and for *S. cyaneus* laccase (valid from pH 4.1 to 5.6, R^2: 0.995): $f_{4.5} = 0.699\ \mathrm{pH}^3 - 10.036\ \mathrm{pH}^2 + 46.829\ \mathrm{pH} - 70.201$.

Results – complementary information

2. Fitting of a bi-exponential model to laccase activity stability

The results of the laccase stability tests were fitted with a bi-exponential equation able to model various mechanisms of enzyme inactivation (Eq. 1) (Aymard and Belarbi 2000) by non-linear least squares regression using Matlab (MathWorks, USA), with A_0 and A_t the activity at time 0 and at incubation time t respectively, a and b the pre-exponential factors, and k_1 and k_2 the apparent first order rate constants:

$$\frac{A_t}{A_0} = a\,\exp(-k_1\,t) + b\,\exp(-k_2\,t). \quad \text{(Eq. 1)}$$

The results of the fitting, the best-fit coefficients of the model and the estimated half-life of laccase at different pH are presented in Table S1. In pure water (both enzymes) and at pH 9 for L_{Sc}, the inactivation followed a simple exponential decay, k_1 and k_2 being equal (Table S1). Except for pH 5, 6 and 7 for L_{Sc} where the time series were too short to have confidence in the fitted model, a bi-exponential model was necessary to reproduce the behaviour observed.

Table S1 Best-fit set of coefficients of the bi-exponential model (Eq. 1) fitted to the laccase stability results and calculated half-life at different pH values

S. cyaneus	pH 3	pH 4	pH 5	pH 6	pH 7	pH 8	pH 9	Pure H_2O
R^2	1.000	0.993	0.985	0.990	0.883	0.997	0.955	0.939
a	0.132	0.351	0.509	0.503	0.502	0.175	0.426	0.428
k_1 [d^{-1}]	7.581	2.035	0.674	0.444	0.098	0.007	0.008	0.284
b	1.641	0.614	0.509	0.504	0.506	0.806	0.561	0.792
k_2 [d^{-1}]	915.486	18.518	0.674	0.444	0.098	0.123	0.008	0.284
**$t_{1/2}$ [d]**	**0.0016**	**0.063**	**1.1**	**1.6**	**7.1**	**7.2**	**81.9**	**3.1**
T. versicolor	pH 3	pH 4	pH 5	pH 6	pH 7	pH 8	pH 9	Pure H_2O
R^2	0.998	0.997	1.000	0.999	0.997	0.994	0.992	0.994
a	0.653	0.038	0.149	0.682	0.791	0.807	0.736	0.531
k_1 [d^{-1}]	0.591	0.012	0.038	0.030	0.010	0.010	0.012	0.132
b	0.343	0.930	1.013	0.388	0.241	0.244	0.233	0.637
k_2 [d^{-1}]	36.717	0.230	0.170	0.134	0.133	0.256	0.382	0.132
**$t_{1/2}$ [d]**	**0.45**	**3.0**	**5.8**	**14.4**	**47.3**	**47.3**	**33.6**	**6.4**

R^2: coefficient of determination of the fitting, a and b: pre-exponential factors, k_1 and k_2: apparent first-order rate constants [d^{-1}], and $t_{1/2}$: calculated half-life of the laccase [d]

3. Fitting of a variable order reaction model to the oxidation of micropollutants with laccase

The results (residual concentrations) of the micropollutant oxidation experiment were fitted by non-linear least squares regression with a variable order reaction model (two coefficients, Eq. 2), as proposed by Margot et al. (2013), taking an initial concentration C_0 of 1 (arbitrary units as the initial concentration was always constant). C_t is the residual concentration after a reaction time t, x the order of the reaction, and k the apparent variable order rate constant:

$$\frac{C_t}{C_0} = \left(\frac{d}{k\, t\, C_0^{1/d} + d} \right)^d \quad with \quad d = \frac{1}{x - 1} \quad \forall\, x \neq 1 \qquad \text{(Eq. 2)}$$

The results of the fitting, the best coefficients of the model and the estimated half-life of the pollutants at different pH values are presented in Table S2. The order of reaction varied mainly between 1 and 3, as observed also by Margot et al. (2013).

Table S2 Best-fit coefficients of the variable order reaction model for the degradation of bisphenol A, diclofenac and mefenamic acid and their respective half-lives at different pH values

	**Bisphenol A**		**Diclofenac**		**Mefenamic acid**		
	S. cyaneus	*T. versicolor*	*S. cyaneus*	*T. versicolor*	*S. cyaneus*	*T. versicolor*	Control
	**pH 5**		**pH 5**		**pH 5**		
R^2	0.997	0.997	0.985	0.993	0.992	0.999	0.996
x	1.596	2.167	2.547	1.986	1.321	2.696	2.936
k	3.936	7.623	0.925	2.763	7.733	339.154	34.015
**$t_{1/2}$ [h]**	**5.2**	**3.4**	**32.2**	**8.6**	**2.4**	**0.09**	**1.0**
	**pH 6**		**pH 6**		**pH 6**		
R^2	0.993	0.998	0.982	0.999	0.997	1.000	0.969
x	0.998	2.964	2.175	1.729	1.814	1.437	2.382
k	2.553	233.949	0.469	9.868	1.667	23.333	0.124
**$t_{1/2}$ [h]**	**6.5**	**0.15**	**54.8**	**2.2**	**13.4**	**0.83**	**225**
	**pH 7**		**pH 7**		**pH 7**		
R^2	0.995	0.994	0.980	0.999	0.993	0.998	
x	0.984	1.190	3.120	1.270	4.014	1.438	
k	1.566	3.939	0.251	1.402	0.204	0.741	
**$t_{1/2}$ [h]**	**10.6**	**4.5**	**151**	**13.0**	**276**	**26.2**	

R^2: coefficient of determination of the fitting, x: order of the reaction, k : apparent variable-order rate constant, and $t_{1/2}$: calculated half-life of the micropollutants [h]

4. Evolution of extracellular laccase activity and biomass in *S. cyaneus* cultures

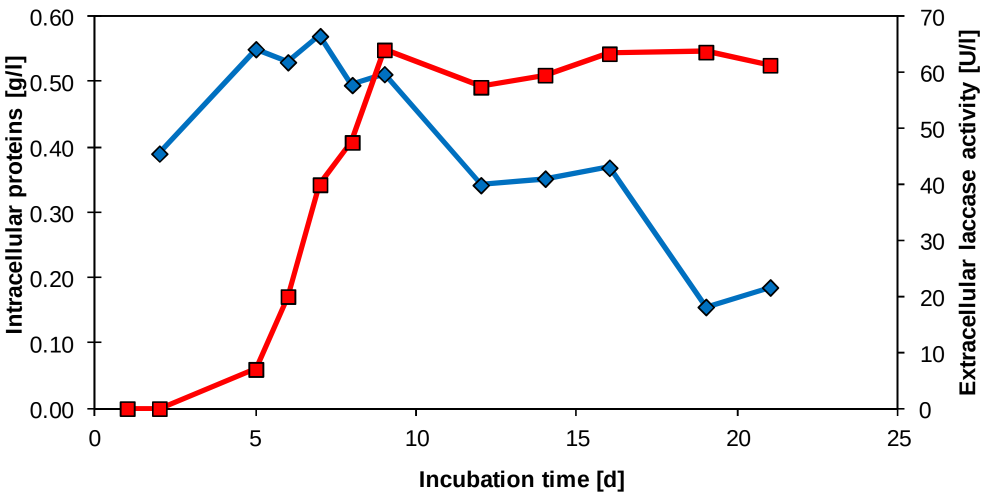

Fig. S2 Evolution of the extracellular laccase activity (■, right axis) and intracellular protein content (♦, left axis, indicator of the biomass) of *S. cyaneus* culture (in modified ISP9 medium with soy flour 10 g l^{-1}). Intracellular proteins were measured with the Bio-Rad DC protein assay kit in the supernatant of pre-washed cells, lyzed by sonication (15 pulses of 3 s at 100 W)

5. Characterization of the commercial laccase preparation from *Trametes versicolor* from Sigma

The commercially available laccase preparation from *Trametes versicolor* obtained from Sigma (Ref. 38429) was analyzed by separating the proteins of 5 µl of concentrated laccase solutions (40 and 5 g l^{-1}) by sodium dodecylsulfate polyacrylamide (12%) gel electrophoresis (SDS-PAGE), following Sambrook et al. (1989). The SDS-PAGE was done with and without 10 min boiling of the proteins. Prior to staining the proteins with Coomassie brilliant blue, one of the duplicate gels was incubated in acetate buffer 100 mM, pH 4.5, with 0.5 mM ABTS to detect the laccase activity.

As presented in Fig. S3, the commercially available laccase preparation contains a mixture of different proteins, from 17 to ~80 kDa, with a major band around 66 kDa, which corresponds approximately to the reported mass of the best-characterized *T. versicolor* laccase isoenzymes (Bourbonnais et al. 1995; Moldes et al. 2004). Similar protein bands from this laccase preparation were also observed by Wang et al. (2012). Despite the denaturing properties of the SDS gel, laccase activity was observed in at least two distinct bands in the gel with unboiled samples, around 40 kDa and 66-70 kDa, suggesting the presence of at least two enzymes with laccase activity in the preparation. The 40 kDa protein showed lower intensity with Coomassie staining but had high laccase activity, suggesting that this protein is thus either very active or more resistant to denaturation than the 66-70 kDa protein. These data show clearly that the commercially available laccase preparation contains a mixture of different proteins, several of which displaying laccase activity.

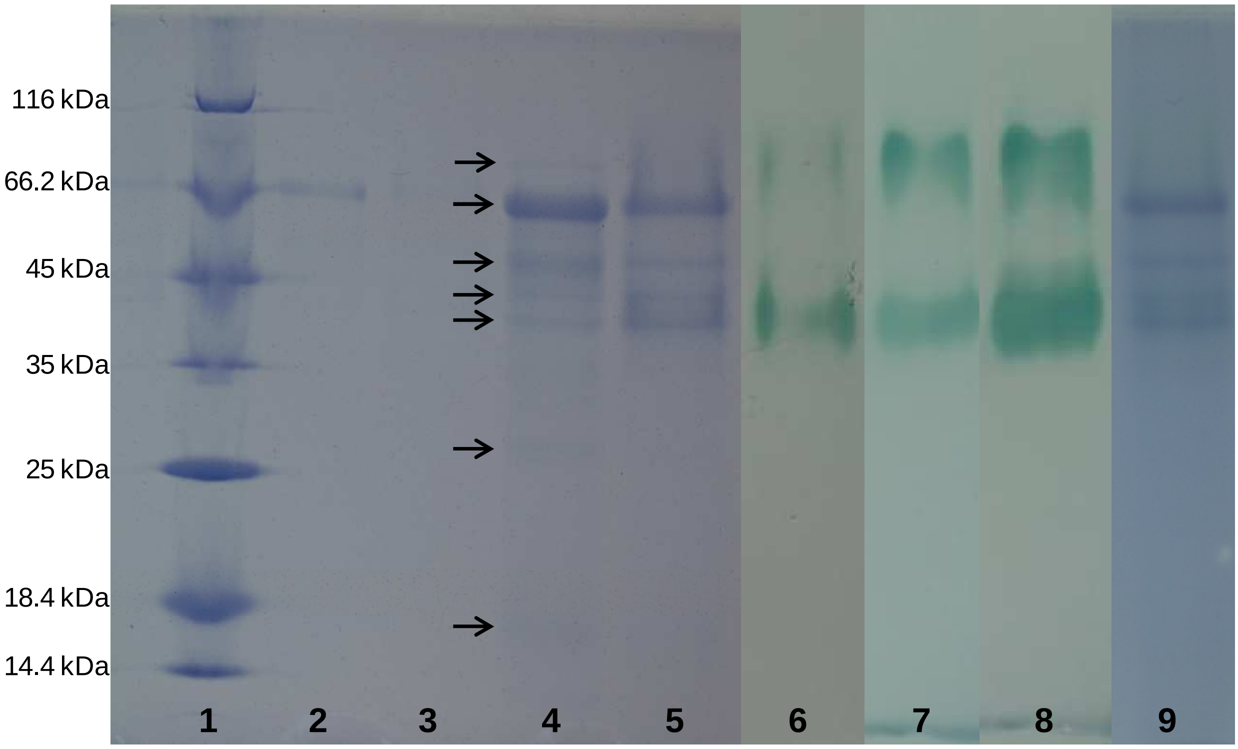

Fig. S3 SDS-PAGE of commercially available laccase preparation from *T. versicolor* from Sigma (Ref. 38429). Lanes 1-5 and lane 9: Coomassie staining; Lanes 6-8: laccase activity with ABTS (0.5 mM in acetate buffer 100 mM pH 4.5). Lane 1: protein ladder (Fermentas); Lanes 2 and 3: 125 µg boiled and unboiled laccase, respectively; Lanes 4 and 5: 1 mg of boiled and unboiled laccase, respectively; Lanes 6-8: 1 mg of unboiled laccase recorded after increasing incubation time in ABTS solution (in green); Lane 9: same lane as 6-8 after additional Coomassie staining. Arrows: bands of proteins present in the commercially available laccase preparation

6. Comparison of "commercial" versus "in house-produced" *Trametes versicolor* laccases

Trametes versicolor is known to produce two main laccase isoenzymes with slightly different kinetic properties (Moldes and Sanromán 2006). The proportion of these two isoenzymes is reported to change depending on the growth substrate, especially in case of addition of lignocellulosic material (Moldes et al. 2004). Thus, the commercially available laccase preparation from *Trametes versicolor* obtained from Sigma (Ref. 38429) may not be fully representative of the laccase produced in a biofilter system with wood chips as the substrate/support. To assess if there was significant difference on micropollutant oxidation kinetics by both laccase preparations, we compared the oxidation kinetics of three micropollutants, bisphenol A (BPA), diclofenac (DFC) and mefenamic acid (MFA), by either the commercial laccase (from Sigma) or laccase produced on wood substrate.

Laccase production on wood substrate

T. versicolor was grown in a glass column (used as a trickling filter) on oak wood by addition of mycelium inoculum on moistened autoclaved wood chips. Once the wood was completely colonized by the mycelium, a synthetic wastewater containing micro and macro nutrients (Borràs et al. 2008), 4 g l^{-1} of glucose and 10 mM MOPS buffer (pH 7), was filtered through the colonized wood chips as in a trickling filter. The water was continuously recirculated and laccase activity was regularly monitored. After 3 d of recirculation, when the activity reached 2000 U l^{-1}, the solution was filtrated at 0.22 µm and used as "produced on-site" laccase preparation.

Micropollutants oxidation essay

Oxidation of a mixture of three micropollutants, BPA, DFC and MFA, at 20 mg l^{-1}, was conducted as described in the main manuscript, in 20 mM citrate-phosphate buffer at two different pH values: 5.8 and 6.8. "Produced" or "commercial" laccase preparations were added to the reaction mixture at the same initial activity of 570 to 580 U l^{-1}. To have similar reaction mixture compositions between both experiments, the same amount of "produced" laccase preparation was also added, after heat inactivation, to the solution containing commercial laccase. Indeed, the "produced" preparation contained some organic substances leached from the wood substrate that may have an effect on the oxidation kinetics. Micropollutant concentration was then followed during 10 h as described in the main manuscript. Duplicate experiments were conducted at 25°C.

Results

As presented in Fig. S4, for both pH values tested, both laccase preparations had very similar oxidation kinetics for BPA and MFA, with no significant difference in the degradation rates. For DFC, the commercial laccase preparation was slightly less efficient at both pH values than the "produced" one, but with less than 10% difference in the removal rates. These very similar oxidation kinetics observed at two different pH values on three different micropollutants show that the commercial laccase preparation is representative, for micropollutant oxidation, of the laccase produced on wood substrate in a trickling filter.

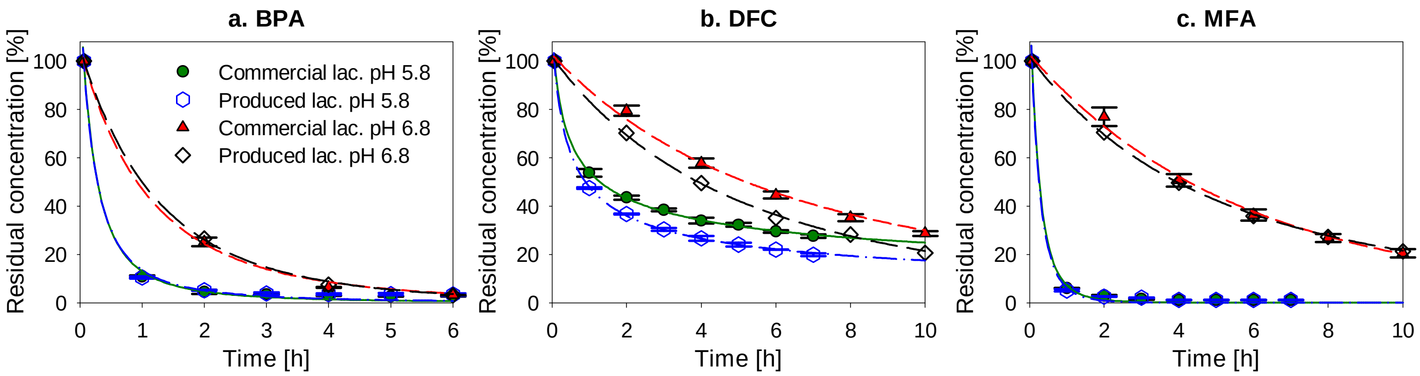

Fig. S4 Residual concentrations of (a) bisphenol A (BPA), (b) diclofenac (DFC) and (c) mefenamic acid (MFA), as a function of the reaction time with commercial (from Sigma) (●,▲) and produced (on wood substrate) (○,◊) laccase preparations from *T. versicolor*, at pH 5.8 and 6.8, 570-580 U l^{-1}, 25°C. Average and values (error bars) of duplicate. Lines: variable order reaction model fitted to the data

7. Evolution of pH during the laccase stability test - Example for the incubation at an initial pH of 4

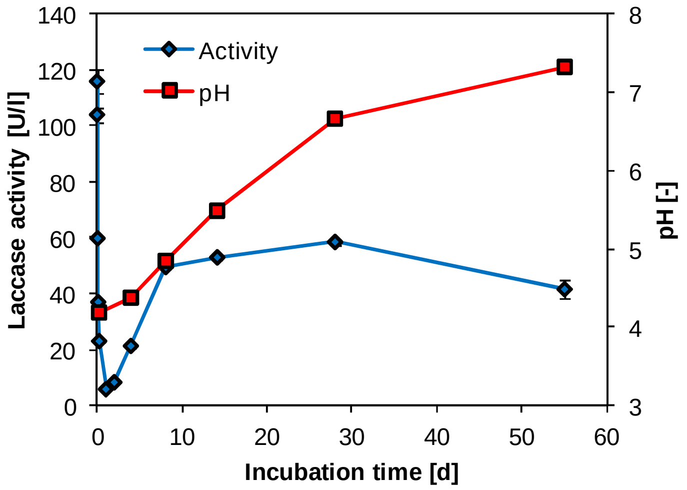

Fig. S5 Evolution of *S. cyaneus* laccase activity incubated at 25°C in citrate-phosphate buffer at an initial pH value of 4 and evolution of the pH due to bacterial growth. Average and values of duplicates (difference in the duplicate pH values lower than 0.06 unit)

8. Production of laccase activity in treated wastewater by *T. versicolor*

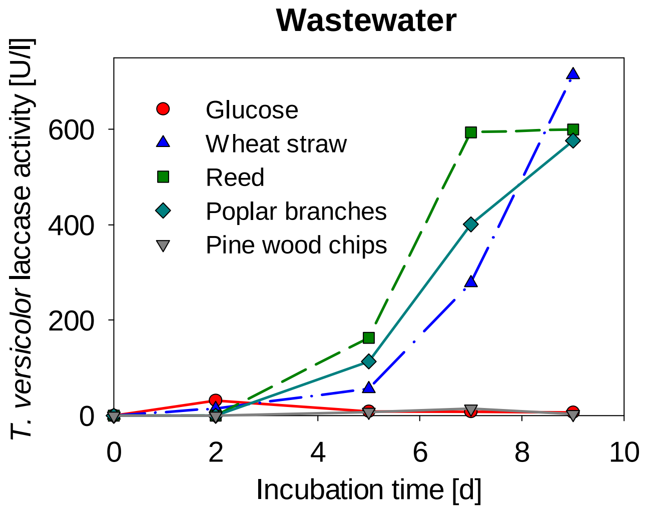

Fig. S6 Evolution of laccase activity in the supernatant of *T. versicolor* cultures (25°C, 140 rpm, pH 5-6.8) in sterile treated (activated sludge without nitrification) municipal wastewater with diverse substrates: glucose (10 g l^{-1}), wheat straw pieces (47 g l^{-1}), reed pieces (*Phragmites australis*, 153 g l^{-1}), poplar (*Populus* spp., 124 g l^{-1}) branches with the bark and pine wood chips (without bark, 123 g l^{-1})

9. Identification of one laccase candidate from *S. cyaneus* culture supernatant

Extracellular crude enzyme preparation of *S. cyaneus* culture supernatant was concentrated 80 times by ultrafiltration as described in the manuscript, and then separated by sodium dodecylsulfate polyacrylamide (12%) gel electrophoresis (SDS-PAGE) following Sambrook et al. (1989). A protein band around 75 kDa corresponding to the predicted *S. cyaneus* laccase molecular mass (Arias et al. 2003) was analysed by mass spectrometry (MS) after trypsin digestion and compared to profiles of peptides generated from available *Streptomyces* genomes and from the deposited *S. cyaneus* laccase sequence (GenBank HQ857207). This analysis was performed by the PCF laboratory (EPFL, Switzerland).

MS analysis of the excised 75 kDa protein band obtained after concentrating *S. cyaneus* culture supernatant showed a profile matching with nine unique peptides (20% coverage) of the deposited laccase sequence (GenBank HQ857207). This latter protein sequence shows 84% amino acid sequence identity with the phenoxazinone synthase (PHS) of *S. antibioticus* (Hsieh and Jones 1995) (see sequence alignment below). This laccase, along with several other *Streptomyces* proteins, form a distinct multi-copper oxidase family either classified as laccase (EC 1.10.3.2) or phenoxazinone synthase (EC 1.10.3.4). Functional differentiation between these two classes is unclear (Le Roes-Hill et al. 2009). The reported *S. cyaneus* laccase shows 33% sequence identity with the well-characterized CotA laccase of *Bacillus subtilis* (GenBank AAB62305) (Martins et al. 2002), and only very limited sequence identity with the EpoA laccase of *S. griseus* (GenBank BAB64332) (Endo et al. 2003) or the laccase of *T. versicolor* (GenBank CAA77015) (Fig. S7). Despite its relatively low sequence homology with other well-characterized laccases, the structure and active site configuration of *S. antibioticus* PHS, a close parent of *S. cyaneus* laccase, is reported to be very similar to other laccases, with three conserved cupredoxin-like domains, T1 (type 1 Cu centre) where the substrate oxidation takes place, and a trinuclear Cu cluster T2 and T3 where the electrons are transferred and where the reduction of oxygen to water take place (Enguita et al. 2003; Smith et al. 2006). Thus, similar catalytic mechanisms for these enzymes are expected. It is, however, important to mention that the *S. cyaneus* laccase activity was measured in the culture supernatant, which might also contain several other laccases not yet identified or reported in databases.

10. Comparison of sequences of *S. cyaneus* laccase and *S. antibioticus* phenoxazinone synthase

The amino acids sequences of *S. cyaneus* laccase (*Scy*-laccase, GenBank HQ857207) and *S. antibioticus* phenoxazinone synthase (*San*-PhsA, GenBank AAA86668) are presented below. A high sequence identity (84%, in black) exists between both enzymes. The residues that bind the different copper atoms (active sites T1 and T2, mononuclear, and T3, binuclear) are presented in color, following Smith et al.(2006). A fifth copper centre, not present in other laccases, was identified. This copper is thought to participate in the stability of the structure but not in the oxidation mechanisms (Smith et al. 2006). The proteins are separated into three main domains, presented with the dashed lines with different colours.

--- Domain 1 (37-236) --- Domain 2 (237-411) --- Domain 3 (439-628)

type 1 copper-1 binding residues type 3 copper-2 binding residues type 3 copper-3 binding residues

type 2 copper-4 binding residues New type 2 copper-5 binding residues

```
San-PhsA       1 MIEQSDDRIDPIDGVLADGVLADDVLAKEREQAPAPGELTPFAAPLTVPPVLRPASDEVT
Scy-laccase    1 ----MTDIIERLT----------DSDGKPEEEQLGTGELTPYTAPLPVPPVLRPASDDVL

San-PhsA      61 RETEIALRPTWVRLHPQLPPTLMWGYDGQVPGPTIEVRRGQRVRIAWTNRIPKGSEYPVT
Scy-laccase   47 HETEIALRPAWVRLHPQLPPTLMWGYDGQVPGPTIEVRRGQRVRIAWTNRIPKDSEYPVT

San-PhsA     121 SVEVPLGPPGTPAPNTEPGRGGVEPNKDVAALPAWSVTHLHGAQTGGGNDGWADNAVGFG
Scy-laccase  107 SVEVPLRTDGRPQSTTEPGREGVEPNKDVAALPAWSVTHLHGAQTGGGNDGWADNAVGFG

San-PhsA     181 DAQLSEYPNDHQATQWWYHDHAMNITRWNVMAGLYGTYLVRDDEEDALGLPSGDREIPLL
Scy-laccase  167 DAQLSEYPNDHQAVQWWYHDHAMNITRWNVMTGLYGTYLVRDDEEDALHLPCGEREIPLL

San-PhsA     241 IADRNLDTDEDGRLNGRLLHKTVIVQQSNPETGKPVSIPFFGPYTTVNGRIWPYADVDDG
Scy-laccase  227 LADRNLDTDEDGRLNGRLLHKTLIVQQQNPETGKPVSIPFSGPYNTVNGRIWPYADVDDA

San-PhsA     301 WYRLRLVNASNARIYNLVLIDEDDRPVPGVVHQIGSDGGLLPRPVPVDFDDTLPVLSAAP
Scy-laccase  287 WYRFRLVNASNARIYDLVLVDEDDNPVPGIVHQIGSDGGLLPRPVPVDFDGALPTLTAAP

San-PhsA     361 AERFDLLVDFRALGGRRLRLVDKGPGAPAGTPDPLGGVRYPEVMEFRVRETCEEDSFALP
Scy-laccase  347 AERFDLLVDFRGLAGRRLRLVNKGRNQPPGVSDPAGDVRYPAVMEFRVRESCETDTFELP

San-PhsA     421 EVLSGSFRRMSHDIPHGHRLIVLTPPGTKGSGGHPEIWEMAEVEDPADVQVPAEGVIQVT
Scy-laccase  407 EVLSGSFRRLTHDIEHGHRLIVLTPPATKGGGGHPEIWEMTEVQNPGDIQVPTEGVIQVT

San-PhsA     481 GADGRTKTYRRTAATFNDGLGFTIGEGTHEQWTFLNLSPILHPMHIHLADFQVLGRDAYD
Scy-laccase  467 GADGKTKTYRRTARTFNDGLGFTIAEGSHEQWSFLNLAPIVHPMHIHLADFQLLGRDAYD

San-PhsA     541 ASGFDLALGGTRTPVRLDPDTPVPLAPNELGHKDVFQVPGPQGLRVMGKFDGAYGRFMYH
Scy-laccase  527 VSGFDPAIGGTRSPIRHDAGTTIPLAPNELGHKDVFRVPGNQILRVMGKFDGAYGRFMYH

San-PhsA     601 CHLLEHEDMGMMRPFVVMPPEALKFDHGGAHG---GHGEGHTG
Scy-laccase  587 CHLLEHEDMGMMRPFVVMPPEALKFDHGAGHGGHDGHGAGHTG
```

11. Phylogenetic tree between different multicopper oxidases

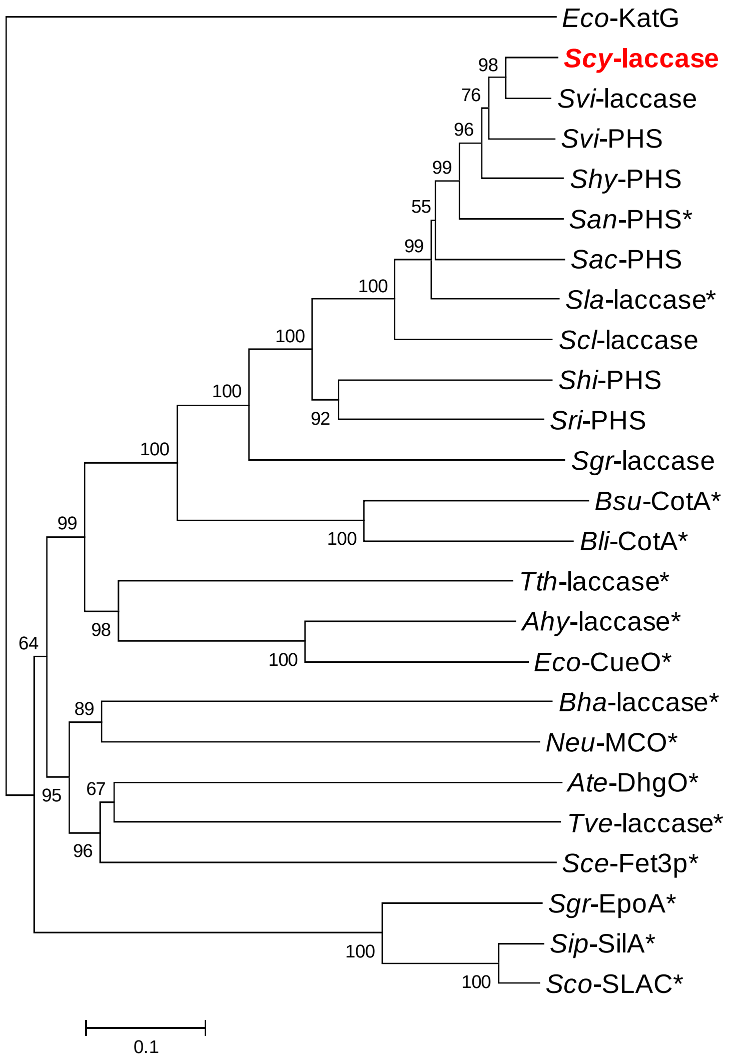

Fig. S7 Sequence likelihood analysis of laccases. The identified laccase of *Streptomyces cyaneus* (*Scy*-laccase, in red, GenBank HQ857207) was compared (using ClustalX and MEGA4) to characterized multicopper oxidases (indicated by *), and to protein sequences found in databases which show a minimal sequence identity of 50% (with >90% sequence length coverage)

<u>Legend for microbial species and sequence references</u>: ***Eco*-KatG**: *Escherichia coli* (GenBank: YP_491509), used here to root the tree; ***Svi*-PHS**: *Streptomyces viridochromogenes* phenoxazinone synthase (WP_003993803); ***Shy*-PHS**: *Streptomyces hygroscopicus* (YP_006248289); ***San*-PHS**: *Streptomyces antibioticus* (AAA86668) (Smith et al. 2006); ***Sac*-PHS**: *Streptomyces acidiscabies* (WP_010360990); ***Sla*-laccase**: *Streptomyces lavendulae* (BAC16804) (Suzuki et al. 2003); ***Scl*-laccase**: *Streptomyces clavuligerus* (WP_003957540); ***Shi*-PHS**: *Streptomyces himastatinicus* (WP_009715166); ***Sri*-PHS**: *Streptomyces rimosus* (WP_004571981); ***Sgr*-laccase**: *Streptomyces griseus* (YP_001821963); ***Bsu*-CotA**: *Bacillus subtilis* (AAB62305) (Martins et al. 2002); ***Bli*-CotA**: *Bacillus licheniformis* (YP_077905) (Koschorreck et al. 2008); ***Tth*-laccase**: *Thermus thermophilus* (YP_005339) (Miyazaki 2005); ***Ahy*-laccase**: *Aeromonas hydrophila* (ACX47357) (Wu et al. 2010); ***Eco*-CueO**: *Escherichia coli* (YP_488426) (Grass and Rensing 2001); ***Bha*-laccase**: *Bacillus halodurans* (AAP57087) (Ruijssenaars and Hartmans 2004); ***Neu*-MCO**: *Nitrosomonas Europaea* multicopper oxidase (PDB 3G5W) (Lawton et al. 2009); ***Ate*-DhgO**: *Aspergillus terreus* dihydrogeodin oxidase (BAA08486) (Huang et al. 1995); ***Tve*-laccase**: *Trametes versicolor* (CAA77015) (Jönsson et al. 1995); ***Sce*-Fet3p**: *Saccharomyces cerevisiae* (CAA89768) (Stoj et al. 2007); ***Sgr*-EpoA**: *Streptomyces griseus* (YP_001822531) (Endo et al. 2002); ***Sip*-SilA**: *Streptomyces ipomoeae* (ABH10611) (Molina-Guijarro et al. 2009); ***Sco*-SLAC**: *Streptomyces coelicolor* small laccase (CAB45586) (Machczynski et al. 2004).

12. References

Arias ME, Arenas M, Rodríguez J, Soliveri J, Ball AS, Hernández M (2003) Kraft pulp biobleaching and mediated oxidation of a nonphenolic substrate by laccase from *Streptomyces cyaneus* CECT 3335. Appl Environ Microbiol 69(4):1953-1958

Aymard C, Belarbi A (2000) Kinetics of thermal deactivation of enzymes: A simple three parameters phenomenological model can describe the decay of enzyme activity, irrespectively of the mechanism. Enzyme Microb Technol 27(8):612-618

Borràs E, Blánquez P, Sarrà M, Caminal G, Vicent T (2008) *Trametes versicolor* pellets production: Low-cost medium and scale-up. Biochem Eng J 42(1):61-66

Bourbonnais R, Paice MG, Reid ID, Lanthier P, Yaguchi M (1995) Lignin oxidation by laccase isozymes from *Trametes versicolor* and role of the mediator 2,2'-azinobis(3-ethylbenzthiazoline-6-sulfonate) in kraft lignin depolymerization. Appl Environ Microbiol 61(5):1876-1880

Endo K, Hayashi Y, Hibi T, Hosono K, Beppu T, Ueda K (2003) Enzymological characterization of EpoA, a laccase-like phenol oxidase produced by *Streptomyces griseus*. J Biochem 133(5):671-677

Endo K, Hosono K, Beppu T, Ueda K (2002) A novel extracytoplasmic phenol oxidase of *Streptomyces*: Its possible involvement in the onset of morphogenesis. Microbiology 148(6):1767-1776

Enguita FJ, Martins LO, Henriques AO, Carrondo MA (2003) Crystal structure of a bacterial endospore coat component: A laccase with enhanced thermostability properties. J Biol Chem 278(21):19416-19425

Grass G, Rensing C (2001) CueO is a multi-copper oxidase that confers copper tolerance in *Escherichia coli*. Biochem Biophys Res Commun 286(5):902-908

Hsieh CJ, Jones GH (1995) Nucleotide sequence, transcriptional analysis, and glucose regulation of the phenoxazinone synthase gene (*phsA*) from *Streptomyces antibioticus*. J Bacteriol 177(20):5740-5747

Huang KX, Fujii I, Ebizuka Y, Gomi K, Sankawa U (1995) Molecular cloning and heterologous expression of the gene encoding dihydrogeodin oxidase, a multicopper blue enzyme from *Aspergillus terreus*. J Biol Chem 270(37):21495-21502

Jönsson L, Sjöström K, Häggström I, Nyman PO (1995) Characterization of a laccase gene from the white-rot fungus *Trametes versicolor* and structural features of basidiomycete laccases. Biochim Biophys Acta 1251(2):210-215

Koschorreck K, Richter SM, Ene AB, Roduner E, Schmid RD, Urlacher VB (2008) Cloning and characterization of a new laccase from *Bacillus licheniformis* catalyzing dimerization of phenolic acids. Appl Microbiol Biotechnol 79(2):217-224

Lawton TJ, Sayavedra-Soto LA, Arp DJ, Rosenzweig AC (2009) Crystal structure of a two-domain multicopper oxidase: implications for the evolution of multicopper blue proteins. J Biol Chem 284(15):10174-10180

Le Roes-Hill M, Goodwin C, Burton S (2009) Phenoxazinone synthase: What's in a name? Trends Biotechnol 27(4):248-258

Machczynski MC, Vijgenboom E, Samyn B, Canters GW (2004) Characterization of SLAC: A small laccase from Streptomyces coelicolor with unprecedented activity. Protein Sci 13(9):2388-2397

Margot J, Maillard J, Rossi L, Barry DA, Holliger C (2013) Influence of treatment conditions on the oxidation of micropollutants by *Trametes versicolor* laccase. N Biotechnol 30(6):803-813

Martins LO, Soares CM, Pereira MM, Teixeira M, Costa T, Jones GH, Henriques AO (2002) Molecular and biochemical characterization of a highly stable bacterial laccase that occurs as a structural component of the *Bacillus subtilis* endospore coat. J Biol Chem 277(21):18849-18859

Miyazaki K (2005) A hyperthermophilic laccase from *Thermus thermophilus* HB27. Extremophiles 9(6):415-425

Moldes D, Lorenzo M, Sanromán MA (2004) Different proportions of laccase isoenzymes produced by submerged cultures of *Trametes versicolor* grown on lignocellulosic wastes. Biotechnol Lett 26(4):327-330

Moldes D, Sanromán MA (2006) Amelioration of the ability to decolorize dyes by laccase: Relationship between redox mediators and laccase isoenzymes in *Trametes versicolor*. World J Microbiol Biotechnol 22(11):1197-1204

Molina-Guijarro JM, Pérez J, Muñoz-Dorado J, Guillén F, Moya R, Hernández M, Arias ME (2009) Detoxification of azo dyes by a novel pH-versatile, salt-resistant laccase from *Streptomyces ipomoea*. Int Microbiol 12(1):13-21

Ruijssenaars HJ, Hartmans S (2004) A cloned Bacillus halodurans multicopper oxidase exhibiting alkaline laccase activity. Appl Microbiol Biotechnol 65(2):177-182

Sambrook J, Fritsch EF, Maniatis T (1989) Molecular cloning: A laboratory manual. Cold Spring Harbor Laboratory Press, Cold Spring Harbor, New York

Smith AW, Camara-Artigas A, Wang M, Allen JP, Francisco WA (2006) Structure of phenoxazinone synthase from *Streptomyces antibioticus* reveals a new Type 2 copper center. Biochemistry 45(14):4378-4387

Stoj CS, Augustine AJ, Solomon EI, Kosman DJ (2007) Structure-function analysis of the cuprous oxidase activity in Fet3p from *Saccharomyces cerevisiae*. J Biol Chem 282(11):7862-7868

Suzuki T, Endo K, Ito M, Tsujibo H, Miyamoto K, Inamori Y (2003) A thermostable laccase from *Streptomyces lavendulae* REN-7: Purification, characterization, nucleotide sequence, and expression. Biosci, Biotechnol, Biochem 67(10):2167-2175

Wang F, Huang W, Guo C, Liu CZ (2012) Functionalized magnetic mesoporous silica nanoparticles: Fabrication, laccase adsorption performance and direct laccase capture from *Trametes versicolor* fermentation broth. Bioresour Technol 126:117-122

Wu J, Kim KS, Lee JH, Lee YC (2010) Cloning, expression in *Escherichia coli*, and enzymatic properties of laccase from *Aeromonas hydrophila* WL-11. J Environ Sci 22(4):635-640
